# Do Domestic Pigs Acquire a Positive Perception of Humans through Observational Social Learning?

**DOI:** 10.3390/ani11010127

**Published:** 2021-01-08

**Authors:** Daniela Luna, Catalina González, Christopher J. Byrd, Rocío Palomo, Elizabeth Huenul, Jaime Figueroa

**Affiliations:** 1 Departamento de Ciencias Animales, Facultad de Agronomía e Ingeniería Forestal, Pontificia Universidad Católica de Chile, Vicuña Mackenna 4860, Macul, Santiago 7820436, Chile; dlunavet@gmail.com (D.L.); cata2403@gmail.com (C.G.); ekhuenul@uc.cl (E.H.); 2 Deparment of Animal Sciences, North Dakota State University, NDSU Dept. 7630, P.O. Box 6050, Fargo, ND 58108-6050, USA; christopher.byrd@ndsu.edu; 3 Departamento de Fomento de la Producción Animal, Facultad de Ciencias Veterinarias y Pecuarias, Universidad de Chile, Santa Rosa 11735, La Pintana, Santiago 8820808, Chile; rocio.palomo@ug.uchile.cl

**Keywords:** human-pig relationship, gentle handling, pig welfare, social learning, social status

## Abstract

**Simple Summary:**

The human-animal relationship is an important component of farm animal welfare. Previous studies have shown that allowing animals to observe the gentle handling of a conspecific reduces fear and increases their affinity toward humans, a phenomenon attributed to observational social learning. This study investigated whether pigs are able to positively perceive and interact with a human after observing a conspecific (i.e., demonstrator pig) receiving long-term gentle handling by a stockperson. We also investigated whether this social learning was biased by the demonstrator’s social rank. Our results show that the observer pigs’ behavior was indicative of a greater affinity toward the stockperson regardless of whether they observed a socially dominant or subordinate demonstrator pig receiving gentle handling. Furthermore, pigs observing the gentle handling of a demonstrator pig exhibited lower physiological stress when they were confronted by the stockperson compared to pigs who received only minimal human contact. This study could have relevance in intensive swine production systems, where stockpersons have limited time to positively interact with the animals. Regular positive handling of a small number of selected pigs could be a useful strategy for reducing fear and stress in large groups of pigs.

**Abstract:**

Farm animals can perceive humans positively by observing another animal being positively handled. This study evaluated whether pigs acquire a positive perception of humans after observing either a high or low socially ranked conspecific receiving gentle handling. Seventy-five 21-week-old pigs were housed in 15 nursery pens (five pigs/pen) and randomly assigned to one of three pen treatments: Dominant Demonstrator Group (DDG), Subordinate Demonstrator Group (SDG) and Control Group (CG). Pigs from DDG and SDG observed a high and low socially ranked conspecific (“demonstrator”), respectively, while the demonstrator received gentle stroking and a sucrose solution for 10 min, twice a day for 5 weeks. Control group pigs received minimal human contact. Following treatment, the behavior and heart rate variability of non-demonstrator pigs were evaluated in response to a stockperson in an open-field test. Pigs from the DDG and SDG contacted the stockperson sooner (*p* < 0.001), spent more time investigating the stockperson (*p* < 0.05), accepted more stroking (*p* < 0.001) and exhibited a lower low/high frequency ratio (*p* = 0.015) compared to the CG. No differences in learning between the pigs from the DDG and SDG were found. These results suggest that pigs can learn to perceive humans positively through observational social learning, regardless of the demonstrator conspecific’s social rank.

## 1. Introduction

The human-animal relationship (HAR) is an important component of farm animals’ welfare [1]. Despite intensive production systems becoming increasingly automated, farm animals are still under human care and interact with stockpeople in different contexts. The nature (positive, negative or neutral) of the interactions between the stockperson and livestock has a strong impact on the animal’s welfare, performance and ease of handling [2]. Animals exposed to regular gentle contact (e.g., stroking/grooming) are less afraid of people and experience lower stress levels [3,4,5] than animals that receive minimal human contact or are negatively handled [6,7,8]. Gentle handling of animals, such as stroking them on a regular basis, talking to them in a soft gentle voice or rewarding them with food, facilitate the development of a positive HAR [4,9,10]. A positive HAR is commonly characterized by a reduced level of stress and fearfulness toward people and a greater affinity toward the human, expressed by an increased motivation to approach and make physical contact [2,3].

In pigs, provision of positive tactile contacts, such as stroking, scratching or brushing [11] and/or the provision of food rewards [8] are strategies that facilitate the development of a positive human-pig relationship, allowing the animal to acquire a conditioned positive perception of the human [12]. Positive perception of humans can, in turn, positively affect the emotional state of pigs [11] and their cognitive abilities [13]. Likewise, a positive perception can improve pig performance parameters such as feed conversion efficiency [14] and daily feed intake [15] during certain productive periods. In consequence, achieving a positive human-pig relationship can be advantageous for both animal and farmer well-being [12]. However, in modern swine production, human-pig interactions have been reduced and animals are exposed to minimal contact with the stockperson. Furthermore, during the production cycle, pigs often experience only neutral or aversive interactions with their caregiver (e.g., during procedures such as castration, vaccination, tail or teeth cutting, transport), making it even more difficult for animals to acquire a positive perception of humans. Therefore, the search for strategies that allow the stockperson to be positively perceived by animals is necessary, considering that provision of a regular gentle handling to all individuals is neither feasible nor practical under intensive production systems [12,16].

Social learning is the capacity of an individual to acquire highly adaptive information from the environment by observation or interaction with a more experienced animal (“demonstrator”), typically a conspecific [16,17]. This learning can be affected (or biased) by social rank of the demonstrator individual, with higher-ranking animals being more effective demonstrators in social transmission of information than their subordinates [18].

The acquisition of a positive perception of humans through social learning has been documented in domestic animals, such as cattle [19], birds [20,21] and horses [22,23], and recently in exotic animals such as guinea pigs [24]. Munksgaard et al. [19] reported that cows that watched their neighbors receiving a gentle handling (i.e., strokes, food reward and a soft gentle voice) showed a lower distance to the handler after observing the handling of their neighbors. In turn, Jones [20] found that, after allowing birds to observe other birds (“demonstrators”) being stroked, the avoidance response toward the human was reduced in “observer” birds, even though they had never received positive tactile contacts.

Most studies about social learning in pigs have focused on feeding behavior [25,26,27]. Figueroa et al. [27] showed that pigs can socially learn to prefer a new flavored feed after a brief interaction with an experienced demonstrator pig that previously consumed the feed. However, no published studies to date have investigated pigs’ social learning abilities regarding the acquisition of a positive perception of the human. In addition, it remains unclear if social learning of the observer animals regarding a positive human-animal relationship is influenced by group social dynamics, specifically by the demonstrator animal social rank. Therefore, the first aim of this study was to determine whether pigs can learn to positively perceive the human through observation of a conspecific demonstrator receiving long-term gentle handling. According to the evidence previously reported, we hypothesized that observer pigs exposed to a demonstrator pig receiving gentle handling would acquire a positive perception of humans through social learning, expressed through behavioral and physiological responses that denote lower stress levels and a greater affinity toward the human compared to animals receiving minimal human contact, as typically experienced in routine care and management under commercial conditions. The second aim of this study was to determine whether learning performance in observer pigs can be affected by the demonstrator social status, elucidating if the dominant conspecifics have a greater effectiveness in information social transmission. We hypothesized that learning performance could be affected by the demonstrator’s social rank, where observer pigs exposed to socially dominant demonstrators could be more attentive to its cues and, therefore, would experience lower stress and exhibit fewer behavioral indicators of fear toward the human than observer pigs exposed to subordinate demonstrators.

## 2. Materials and Methods

The experiments were conducted between October 2019 and November 2019 at the swine experimentation facility at the Centro de Investigación, Innovación Tecnológica y Capacitación para la Industria Porcina Nacional (CICAP), at the Pontificia Universidad Católica de Chile (PUC) in Santiago, Chile. All experimental procedures were approved by the Ethical Committee on Animal Experimentation of the PUC (no. 180928004).

### 2.1. Animals and Housing

A total of 75 castrated male (*n* = 34) and female (*n* = 41) piglets (PIC Genetics) were weaned at 21 days of age and immediately transported from a commercial pig farm to the swine experimental facilities at the CICAP (Day 1; Table 1). On arrival, the pigs were weighed (5.6 ± 0.2 kg) and randomly allocated to 15 nursery pens (1.80 m × 1.28 m × 0.7 m; fully slatted floor) in groups of five pigs considering similar weights between pens (*p* > 0.05). Animals housed in each pen were individually identified by using numbered plastic ear tags of different color. Additionally, pigs were identified with a number on their back using a non-permanent marker pen. Each pen had one feeder with three feeding spaces and an individual water supply. The room was temperature controlled (27.3 ± 2.70 °C) and automatic forced ventilation. The animals were fed with a standard commercial diet (PreStart, Starter 1 and Starter 2 diets; Champion S.A, Santiago, Chile) by the stockperson, according to the nutritional requirements set by the National Research Council [28]. Pigs had ad libitum access to feed and fresh water for the entire experimental period, except during test sessions. Individual animal health status was evaluated daily by visual inspection carried out by the same stockperson and supervised weekly by a veterinarian. All pigs underwent a period of acclimation to the facilities and management conditions during the first post-weaning week (Table 1).

### 2.2. Experimental Set-Up

#### 2.2.1. Determination of Social Dominance Order

Immediately after mixing and housing the pigs, agonistic interactions (AI) in 10 randomly selected pens were registered by continuous video recording over 48 h (see Table 1; Day 1–2). An agonistic interaction was defined as a fight or a displacement with physical contact initiated by one individual including aggressive behavior elements followed by any form of submission performed by the opponent [29,30]. Based on previous studies that evaluated the establishment of social hierarchy in pigs [30,31,32,33], nine hours of focal continuous sampling during the first two days (Day 1: 13:00 h to 22:00 h; Day 2: 9:00 h to 18:00 h; Table 1) after mixing was used for the analysis aimed at establishing the social dominance order inside each pen.

For each pen, one video camera (IR Outdoor Cameras 700tvl 1/3 cmos Sony, SENKO SA, Santiago, Chile) placed on the ceiling in a central position was used to record pig behavior. Afterwards, agonistic behaviors of the animals were analyzed by 10 observers who were previously trained to detect and identify aggressive behaviors in pigs (Day 3–5; Table 1). For each AI, the duration of encounter, the aggressor and who received the aggression, the winner and the loser and the inconclusive fights (i.e., when there was not a clear outcome) were registered by the observers, following the same criteria used by Stukenborg et al. [30]. Subsequently, a Dominance Index (DI) was calculated (Day 6; Table 1) for each animal who was involved in AI, based on the study of Stukenborg et al. [30]. This DI was ranked from −1 (absolute submissive) to +1 (absolute dominant), thus allowing the determination of the most dominant and most subordinate pig inside each pen, who then acted as demonstrator. Behavioral observations were analyzed using the Behavioral Observation Research Interactive Software (BORIS; version 7.8) [34].

#### 2.2.2. Treatments

Each pen was randomly assigned to one of three treatments (five pens/treatment; five pigs/pen): (1) Control Group (CG, *n* = 5 pens): animals from five pens received minimal human contact corresponding to daily routine practices for managing pigs under commercial conditions. These practices consisted of feeding, pen cleaning and health status evaluations, which were performed from outside the pens. Handling was carried out by a stockperson (woman, 1.60 m) who wore green overalls and white boots during the whole experimental period; (2) Dominant Demonstrator Group (DDG, *n* = 5 pens): in addition to receiving the daily care practices previously mentioned, four animals (“observer pigs”) from each of the five pens were exposed to one dominant conspecific (“demonstrator pig”) through a transparent acrylic panel, while it was subjected to gentle human handling according to a standardized procedure (see the “Gentle Handling Procedure” section). Both gentle handling (GH) and care practices were carried out by a single stockperson (woman, 1.50 m) who wore blue overalls and white boots during the whole experimental period; and (3) Subordinate Demonstrator Group (SDG, *n* = 5 pens): four animals from each of five pens were exposed to watch one conspecific subordinate “demonstrator pig” under the same conditions described above for the DDG and carried out by the same stockperson. During the application of the treatment, the stockperson did not physically touch the observer pigs unless it was absolutely necessary.

##### Gentle Handling Procedure

**Figure 1:**
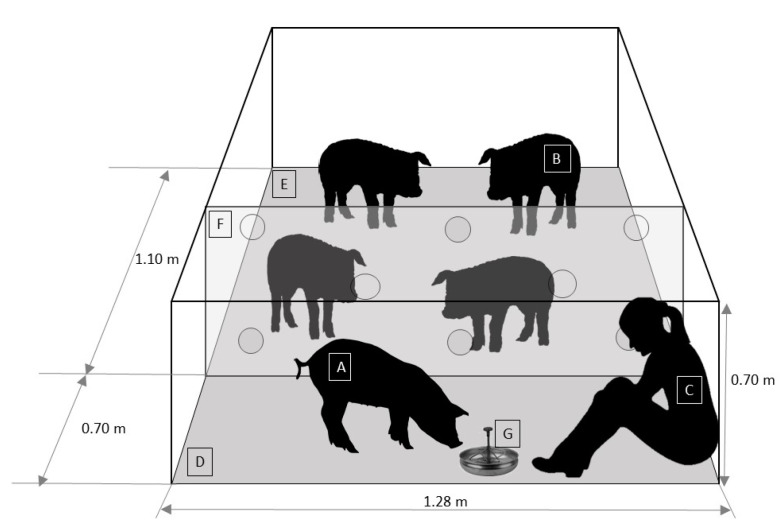
Schematic representation of the pen (front view) during the gentle handling session. A: demonstrator pig (dominant or subordinate), B: observer pig, C: stockperson, D: demonstrator pig handling area, E: observer pig area, F: transparent acrylic panel, G: drinker bowl with 16% sucrose solution.

Demonstrator pigs from the DDG and SDG treatments were subjected to a regular GH procedure on days 8–40 of the experimental procedure (Table 1). The GH procedure was performed for 10 min twice a day (between 9:00 to 11:00 h and 14:00 to 16:00 h; 5 days per week) over a period of 5 weeks. The GH session was standardized as follows: (1) 10 min prior to the start of GH, the pen was temporarily partitioned into two areas (demonstrator pig handling area and observer pig area), through the installation of a transparent acrylic panel (1.26 m × 0.7 m) with eight circular perforations (5 cm diameter) homogeneously distributed (Figure 1). Two disguised experimental personnel with white overalls covering their heads and white mask installed the panel from outside the pen. (2) Then, the stockperson entered the observers´ area, calmly caught the demonstrator pig without touching the rest of the animals, and then released it on the floor of the handling area. (3) When the demonstrator pig was in the handling area, the stockperson entered, sat down on the floor, and remained motionless for 3 s. (4) Then, the stockperson held out their hand toward the demonstrator pig. If the demonstrator pig did not move away, the stockperson tried to touch it. Afterwards, if the demonstrator pig accepted being touched, the stockperson started to stroke it gently with the palm of her hand, from head to back for 7 min, at a rate of 1 stroke every 2 s. If the pig was too restless (e.g., climbing on her shoulders or biting her too hard), then the stockperson gently pushed away the demonstrator pig while also avoiding sudden movements. (5) During the next 3 min, the stockperson offered the demonstrator pig a 16% sucrose solution while stroking it. This solution was used based on the study of Figueroa et al. [35], who found that sucrose palatability in pigs was greater at a 16% concentration. The solution was delivered to the stockperson by one of the disguised experimental personnel from outside the pen. In addition to gentle tactile contact, the stockperson spoke to the demonstrator with a soft voice during the GH session. (6) Once the GH was finished, the stockperson picked up the demonstrator pig and returned it to the observer pigs’ area with their penmates and proceeded to leave the pen. (7) Finally, 10 min after returning the demonstrator pig, the two disguised experimental personnel removed the panel and the drinker bowl with the sucrose solution.

During the GH session, observer pigs had auditory and visual exposure to their conspecific demonstrator. However, olfactory and tactile (snout-snout) contact between observer pigs and demonstrator pig was partially obstructed and was only possible through the panel holes. This was done to avoid interrupting the GH of demonstrator pigs and prevent any type of physical interaction between observer pigs and the stockperson. The animals in each pen had no visual contact with the animals from other pens during the whole experimental procedure but were able to hear and probably smell the pigs from the adjacent pens. Additionally, the animals from CG were isolated from the rest of the treatment groups with a 4 m black curtain installed in the control pens, built out of high-density plastic cloth. Before starting each GH session, the order in which each demonstrator was subjected to GH in the previous session was checked and subsequently re-organized to avoid possible bias associated with the order of GH.

#### 2.2.3. Open-field Arena Test

The behavioral reactions of observer pigs from the CG, DDG and SDG toward their respective stockperson in an unfamiliar environment were evaluated and compared between groups following the GH experimental procedure. Protocols and behavioral observations used in this study were adapted from previous research that assessed the nature of the human-pig relationship/interaction [8,11,14,36]. In addition to behavioral reactions, physiological changes with regards to cardiac activity in response to interaction with the stockperson were evaluated through heart rate variability (HRV) parameters.

##### Behavioral Reactions toward the Stockperson in the Open-Field Arena Test

All pigs were tested individually in an open-field arena over three consecutive days (Days 44–46, Table 1), balancing the groups through the days. The open-field arena was an empty pen (3.11 m × 2.21 m × 0.96 m) with a solid concrete floor located in an adjacent room within the same facility. The arena floor was divided into 20 identical zones (0.55 m × 0.62 m) by white painted lines to record locomotor activity. The pigs were individually transported by two study personnel using a mobile cage (1.5 m × 1.5 m × 0.8 m) equipped with two entrance doors. Personnel wore white overalls and white boots. The pigs in each pen were previously habituated to the transport cage and the arena pen in groups, remaining for 20 min inside the arena (Days 29–31; Table 1). The test order of animals was randomized, and all pigs were returned to their social group (rearing pen) once the individual test was finished. The open-field arena test lasted 7 min and consisted of four phases: (1) The pig was left alone in the pen for 2 min (Phase 1—Habituation to arena: 0–2 min); (2) After the habituation period, the stockperson quietly entered and stood motionless at the middle of the entrance wall for 1 min (Phase 2—Standing stationary person: 2–3 min); (3) Then, the stockperson walked slowly to middle of the right wall of the pen, sat down and remained motionless without interacting with the animal for 2 min (Phase 3—Sitting stationary person: 3–5 min); (4) The stockperson stood up and waited for 5 s before adopting a squatted down posture. Over the next 2 min, the stockperson walked slowly in the squatting position toward the pig with the aim of touching it and stroking it, while speaking with a soft gentle voice. When the stockperson was within one arm´s length of the pig, she slowly bent down to touch it and stroke it. If the pig accepted being touched, the stockperson softly stroked it along the body from head to back, with the palm of her hand. If the pig was too far from the stockperson, then she was allowed to slowly move toward the animal adopting a squatting posture to attempt to touch and stroke them (Phase 4—Moving person: 5–7 min). Finally, after 2 min, the stockperson slowly stood up and left the pen.

##### Behavioral Observations

Behavioral reactions of the pigs to the open-field arena test were video recorded using two video cameras with integrated microphone (DH-HAC-HDW1200EM-A, Zhejiang Dahua Technology, Zhejiang, Hangzhou, China) from two angles in order to capture the entire pen area and the interaction with the human. Behavioral responses indicating fearfulness or affinity toward the stockperson were recorded for each phase of the test. The ethogram used for the behavioral observations is described in Table 2. All animals were individually observed by continuous focal sampling. The behavior observations were analyzed using the BORIS Software [34].

##### Physiological Changes on Cardiac Activity in Response to Interaction with the Stockperson

In order to evaluate the pigs’ emotional response and stress levels during the open-field arena test, heart rate and heart rate variability (HRV) measures were recorded non-invasively using a heart rate monitor, consisting of an electrode belt with built-in transmitter (Polar H10; Polar Electro Oy, Kempele, Finland) and a wristwatch receiver (Polar V800, Polar Electro Oy, Helsinky, Finland). The monitor system allows the storage of interbeat interval (IBI) time series at a sampling rate of 1000 Hz [37]. All pigs underwent a period of habituation to the heart rate monitor and the personnel in charge of its installation before the test. Once a day for two consecutive days (Day 27–28; Table 1), pigs in their home pens were habituated to wearing an elastic belt similar to the heart rate monitor belt used in the experimental procedure. Each habituation session lasted 20 min per pig. Following the habituation period and before the open-field test, each pig underwent a 17-min baseline HRV measurement period (Day 34–35; Table 1), carried out in their home pens. One day before the baseline measurements, the hair behind the forelegs of each pig was cut-off in order to ensure direct contact between the heart rate monitor belt and the pig´s skin. The following day, each pig was manually restrained by one study personnel while another fitted the equipment. Ultrasound transmission gel (Difem Pharma, Difem Laboratorios S.A. Santiago, Chile) was applied at all electrode contact points to improve contact with the skin. The heart rate monitor was put on behind the forelimbs of pig with the transmitter positioned in the left armpit and wrapped with an elastic bandage. Afterwards, the animal was partially separated from its penmates through an acrylic panel placed in their home pen (same panel used in GH procedure), allowing auditory, visual and tactile (snout-snout) contact during baseline HRV measurement. This partial separation was made in order to protect the monitor from potential damage by penmates.

Immediately before starting the open-field test (Day 44–46; Table 1), each pig was fitted with the Polar HR monitor following the same procedure described above. Then, each pig was individually transported by two study personnel to the open-field arena, using a mobile cage partially closed and equipped with two entrance doors. In the open-field arena, one of the study personnel who transported the pig entered the cage through one of the doors, then turned on the monitor and proceeded to free the pig in the experimental arena through the opposite door of the cage.

##### Heart Rate Variability Analysis

After the data collection, each IBI data file was downloaded using the Polar Flow application (Polar Electro Inc.; Lake Success, NY, USA) and then exported to an Excel file (Microsoft^®^ Office 365 version 2011; Microsoft Corporation, Washington, DC, USA) to proceed with the visual inspection and manual edition of data. Percent change was calculated for each IBI with more than a 20% change signaling an error. The errors (artefacts and ectopic beats) were edited manually according to guidelines of Marchant-Forde et al. [37]. Interbeat interval data with a corrected error rate above 5% and segments with more than three consecutive erroneous IBIs were excluded from the analysis [38]. This criterion led to the exclusion of five animals (CG: *n* = 4, SDG: *n* = 1) from HRV analysis.

For baseline HRV analysis, the first 5 min of IBI data recorded from each pig were discarded in order to exclude potential bias associated with previous handling of the animals, and the next 5 min were analyzed. For the HRV analysis during the open-field test, a single data set of consecutive IBIs contained in a period of 5 min was isolated [39]. This period corresponded with the phases where the stockperson was present and interacted with the animal in the arena test (phases 2, 3 and 4), thus representing the overall human-pig interaction.

Heart rate variability parameters in the time and frequency domains were measured. In the time domain, the following parameters were evaluated: (a) mean interval between adjacent heart beats over a period of time (mean RR interval): b) mean heart rate (mean HR); (c) standard deviation of inter-beats intervals (SDNN, which indicates both sympathetic and parasympathetic activity); (d) the root mean square of successive inter-beats intervals (RMSSD, which indicates parasympathetic activity); and (e) the ratio between RMSSD/SDNN (which reflects the overall balance of the autonomic nervous system, where greater values indicate an increased parasympathetic input). In the frequency domain, the following parameters were measured: (a) low frequency band (LF, which reflects both parasympathetic and sympathetic regulation); (b) high frequency band (HF, which reflects parasympathetic regulation); and (c) the ratio between low and high frequency (LF/HF, which reflects the overall balance of the autonomic nervous system, where greater values indicate an increased sympathetic input [38,40]).

Data were analyzed using software for HRV analysis (Kubios HRV Standard, Kubios Oy, Kuopio, Finland). Trend components in each data set were removed by first order differencing. All data used for frequency spectral analysis were re-sampled at 4 Hz to ensure equidistant data points before undergoing fast Fourier transformation. The fast Fourier transform spectrum window width was set to 150 s with a 50% window overlap. The frequency ranges for LF and HF bands were set according to recommendations of Poletto et al. [41].

### 2.3. Statistics

Data analysis was performed using SPSS version 22.0 (IBM Corp, Armonk, NY, USA). For behavioral and physiological analyses, the experimental unit was the pen, and the observational unit was the animal. Data from demonstrator pigs were removed from the analysis in order to not overestimate the results. Behavioral data obtained during the tests were analyzed by phase. Physiological data (heart rate and HRV measures) were analyzed by considering phases 2, 3 and 4 as a single phase. All variables were tested to verify normality (Shapiro-Wilk test) and homogeneity of variance (Levene test).

For behavioral measures, differences between groups (DDG, SGD and CG) were analyzed using a mixed ANOVA, with the treatment (DDG, SDG and CG), sex (male and female) and their interaction as fixed effects. Pen nested within treatment was included as a random effect in each model. An initial analysis showed that there was no significant interaction between treatment and sex for any behavioral measurement during any phase of tests. In consequence, it was not considered in the final models. Additionally, when sex had no significant influence in the models, it was removed, and the data set was re-analyzed. For all models, multiple pairwise comparisons after the mixed ANOVA were made using Least Significant Differences (LSD) [42]. When normality of residuals could not be assumed, some data were transformed to meet assumptions of statistical tests (behavioral data expressed as a percentage were subjected to angular transformation [43]). When the criteria for parametric statistical using transformed data could not be met, the non-parametric Kruskal–Wallis test was used. Post-hoc comparisons of medians were then conducted using the Dun-Bonferroni test. Results are presented as estimated marginal means (E.M.M.) and standard errors means (S.E.M.) of non-transformed data for the mixed ANOVA and as medians and interquartile ranges (25th and 75th percentiles) for the non-parametric Kruskal–Wallis test. Additional data are presented using descriptive statistical (means ± S.E., percentage).

The latencies to enter the area and to first contact with the stockperson were analyzed using the non-parametric Kaplan–Meier survival method [44]. The Kaplan–Meier method uses the individual latencies by considering censored data, which are the non-approaches [8]. To test whether latencies differed statistically between treatments, a log rank significance test was performed. Means and standard errors of latencies were calculated.

The effect of treatment on the number of animals that performed the behaviors of climbing on the stockperson, defecating and urinating was determined by Pearson’s Chi-Square test. Post-hoc comparisons after the Chi-Square test were run using the adjusted standardized residuals with Bonferroni adjusted *p*-value [45].

The physiological measures (HRV parameters) were analyzed as changes compared to the baseline values obtained before the open-field arena test, obtaining relative values (ΔHRV: [46]). Differences between groups were analyzed using Mixed ANOVA, with treatment, sex and their interaction as fixed effects and the pen nested within treatment as a random effect. Mean RR and HR data were analyzed considering locomotor activity as a covariate. No significant interaction between treatment and sex was found for any measurement. Therefore, this interaction was removed from the final models. Multiple pairwise comparisons after Mixed ANOVA were run using LSD. Results are presented as estimated marginal means (E.M.M.) and standard errors means (S.E.M.). For all analysis, a *p*-value of <0.05 was established for significant differences, whereas a *p*-value greater than 0.05 but less than 0.10 was considered a tendency.

## 3. Results

### 3.1. Behavioral Reaction in Open-Field Arena Test

For all the behavioral measures, except for defecation and urination, the values and the effects of treatment are summarized in Table 3. During Phase 1, when pigs remained isolated in arena, no effect of treatment for locomotor activity (F2/11.53 = 0.589, *p* = 0.571), expression of low-pitched vocalizations (F2/61 = 1.746, *p* = 0.183) and high-pitched vocalizations (Kruskal–Wallis, X^2^ = 1.159, *p* = 0.562, df = 2) were found. On average, pigs crossed 36.56 (±1.92) zones and expressed 23.17 (±2.32) grunts. Only 12% of pigs emitted high-pitched vocalizations during Phase 1. No significant effect of sex was found for the behavioral measures during Phase 1 (*p* > 0.10).

**Figure 2:**
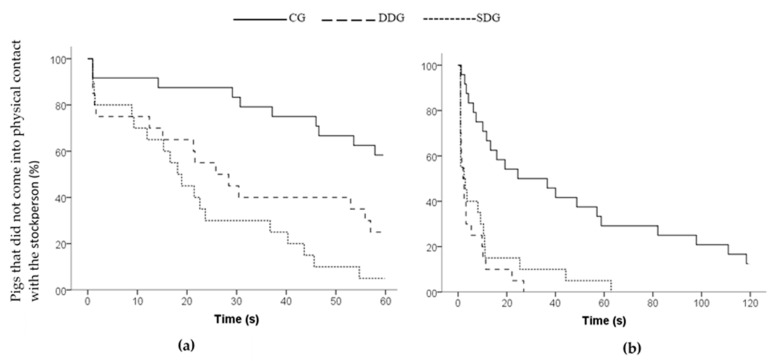
Survivor curve of pigs’ latencies to physical contact with the stockperson during: (**a**) Phase 2 and (**b**) Phase 3 of the open-field arena test. Treatments: Control group (CG), Dominant Demonstrator group (DDG) and Subordinate Demonstrator Group (SDG).

During Phase 2, no differences were found between treatments for locomotion (F2/12.38 = 0.593, *p* = 0.567), low-pitched vocalizations (F2/13.52 = 0.606, *p* = 0.561) and high-pitched vocalizations (Kruskal–Wallis, X^2^ = 3.763, *p* = 0.152, df = 2) when the stockperson entered the arena and remained standing. On average, pigs crossed 9.71 (±1.07) zones and expressed 14.52 (±1.66) and 1.13 (±0.57) low and high-pitched vocalizations, respectively. However, differences were found between treatments in regard to latencies to approach the area around the stockperson (Kaplan–Meier, X^2^ = 18.855, *p* = 0.001, df = 2), to first physical contact with the stockperson (Kaplan–Meier, X^2^ = 19.176, *p* = 0.001, df = 2; Figure 2a) and the time spent looking at the stockperson (F2/12.57 = 4.913, *p* = 0.027). Pigs from the DDG and SDG approached the area and entered into physical contact with the stockperson sooner than the CG pigs (*p* < 0.01), whereas the DDG and SDG pigs did not differ from each other (*p* > 0.05). However, the DDG pigs tended to contact the stockperson later than the SDG pigs (*p* = 0.08). On the other hand, the CG pigs took twice the time than pigs from the other treatments to approach and investigate the stockperson (Table 3), and they spent more time looking at the stockperson compared with pigs from the DDG (*p* = 0.022) and SDG (*p* = 0.017), whereas the DDG and SDG pigs did not differ each other (*p* = 0.909).

In addition, the DDG and SDG pigs spent more time near the stockperson (F2/61 = 6.724, *p* = 0.002) and in contact with her (F2/61 = 6.240, *p* = 0.003) compared to the pigs in the CG. Regarding the frequency of contact, differences were observed between treatments (F2/12.09 = 4.302; *p* = 0.039). Pigs from the DDG and SDG made contact with the stockperson more often than pigs from the CG (*p* < 0.05). However, no differences were found between the DDG and SDG pigs (*p* = 0.575). No significant effect of sex was found for the behavioral measures during phase 2 (*p* > 0.10).

During Phase 3, while the stockperson was sitting and stationary in the arena, treatment affected most of the behaviors of pigs, with the exception of locomotor activity and vocalizations, which only showed a tendency. Compared to the CG pigs, pigs from the DDG and SDG tended to move less between zones (F2/12.40 = 3.62, *p* = 0.058), whereas the DDG and SDG pigs did not differ from each other (*p* = 0.874). In addition, pigs from the DDG and SDG tended to emit a lower number of low-pitched vocalizations (F2/12.79 = 2.763, *p* = 0.100) and high-pitched vocalizations (Kruskal–Wallis, X^2^ = 4.904, *p* = 0.080, df = 2) than the CG pigs.

During Phase 3, pigs from the DDG and SDG showed lower latencies to approach the area (Kaplan–Meier, X^2^ = 25.317, *p* < 0.001, df = 2) and to contact the stockperson (Kaplan–Meier, X^2^ = 25.361, *p* < 0.001, df = 2; Figure 2b) than the GC (*p* < 0.05). Pigs from the DDG and SDG also spent more time near the stockperson (F2/12.56 = 7.380, *p* = 0.008) and in physical contact with the stockperson (F2/12.12 = 6.172, *p* = 0.014) than the GC pigs (*p* < 0.05), while no differences were found between both groups (*p* > 0.10). There was no difference between the DDG and SDG in the time spent looking at the stockperson (*p* = 0.883), and both groups spent less time looking at her than the CG pigs (Kruskal–Wallis, X^2^ = 14.441, *p* = 0.001). Regarding the frequency of contact, no differences were found between treatments (F2/11.90 = 0.026; *p* = 0.975). However, the frequency of contact was affected by sex (F1/55.15 = 4.309; *p* = 0.043). Females showed a greater frequency of contact (4.46 ± 0.53) with the stockperson than males (3.34 ± 0.53). Regarding the frequency of climbing on the stockperson, no differences were observed between treatments (F2/10.91 = 0.551; *p* = 0.592). However, this was affected by sex (F1/55.56 = 8.225; *p* = 0.006), where females showed a greater frequency of climbing (1.77 ± 0.375) on the stockperson than males (0.62 ± 0.38). No differences between treatments were found for the number of pigs climbing on the stockperson (GC: *n* = 10, DDG: *n* = 10, SDG: *n* = 8, Pearson’s Chi-Square = 0.474, *p* = 0.781, df = 2).

During Phase 4 (i.e., the moving stockperson phase), the CG pigs did not behave in the same way as the DDG and SDG pigs. The CG pigs crossed more zones (F2/12.92 = 8.164, *p* = 0.005) and expressed more low-pitched vocalizations (F2/12.85 = 9.292, *p* = 0.003) and high-pitched vocalizations (Kruskal–Wallis, X^2^ = 9.921, *p* = 0.007) than pigs from the other treatments. They also accepted a lower percentage of strokes (F2/11.72 = 25.913, *p* < 0.001) and more attempts were required by the stockperson to complete the first stroke (Kruskal–Wallis, X^2^ = 45.973, *p* < 0.001) compared to the pigs from the DDG and SDG. Moreover, no animals from the control group accepted being stroked at the first attempt, while 70% and 63% of pigs from the DDG and SDG accepted being stroked at the first attempt, respectively. In addition, there was an effect of sex for locomotor activity (F1/59.72 = 4.448, *p* = 0.039), in which females (32.65 ± 1.97) showed a greater movement between zones than males (27.17 ± 2.02).

Regarding the defecating and urinating behaviors, more CG pigs than pigs from the DDG and SDG were observed defecating (GC: *n* = 20, DDG: *n* = 11, SDG: *n* = 10, Pearson’s Chi-Square = 6.303, *p* = 0.043, df = 2) and urinating (GC: *n* = 9, DDG: *n* = 2, SDG: *n* = 2, Pearson’s Chi-Square with Monte Carlo test = 7.008, *p* = 0.039, df = 2) during at least one of the phases (phase 2, 3 or 4) in which the stockperson was present in arena, whereas the DDG and CG did not differ from each other (*p* > 0.10).

### 3.2. Physiological Reaction in the Open-Field Arena Test

For all the physiological measures, the mean values and effects of treatment and sex are summarized in Table 4. No significant differences were found between treatments for mean RR interval (F2/55 = 1.046, *p* = 0.358) and mean heart rate (F2/55 = 0.777, *p* = 0.465). However, RR and HR tended to be affected by sex. Male pigs tended to exhibit lower relative RR (F1/55 = 3.927, *p* = 0.057) and higher relative HR (F1/55 = 2.668, *p* = 0.100) compared to female pigs. Locomotor activity had no significant effect on RR (F1/55 = 0.027, *p* = 0.869) and HR (F1/55 = 0.057, *p* = 0.813).

SDNN did not differ between treatments (F2/10.412 = 1.959, *p* = 0.190). However, differences between sexes were found. Male pigs showed a significantly greater relative value of SDNN (F1/55.06 = 5.713, *p* = 0.020) during the interaction with the stockperson than female pigs. Regarding the RMSSD, there was no effect of treatment (F2/11.81 = 0.721, *p* = 0.506) or sex (F1/55 = 0.786, *p* = 0.379).

The ratio between RMSSD and SDNN (RMSSD/SDNN) was not affected by treatment (F2/11.47 = 0.137 *p* = 0.874) but was affected by sex. Although both sexes showed a decrease compared to their baseline values, male pigs showed a significantly higher decrease of relative values of RMSSD/SDNN ratio (F1/53.85 = 4.757, *p* = 0.034) than female pigs.

LF was affected by treatment (F2/10.239 = 11.048, *p* = 0.003). Control group pigs exhibited greater relative values of LF than pigs from the DDG (*p* = 0.002) and SDG (*p* = 0.002) during the interaction with the stockperson, whereas the DDG and SDG did not differ from each other (*p* = 0.926). Regarding sex, male pigs exhibited greater relative values of LF (F1/54.73 = 11.830, *p* = 0.001) than females. On the other hand, the HF was not affected by treatment (F2/56 = 0.026, *p* = 0.975) or sex (F1/56 = 1.750, *p* = 0.191).

The ratio between low and high frequency power (LF/HF) was affected by treatment (F2/10.35 = 6.423, *p* = 0.015). The relative value of the LF/HF ratio was significantly greater for the CG pigs compared to the DDG pigs (*p* = 0.006) and SDG pigs (*p* = 0.033), whereas the DDG and SDG pigs did not differ from each other (*p* = 0.333). With regard to sex, male pigs showed a significantly higher increase in LF/HF ratio (F1/54.08 = 9.139, *p* = 0.004) than female pigs.

## 4. Discussion

This study investigated the ability of domestic pigs to learn to positively perceive the human through observational social learning. When animals were evaluated in a non-familiar environment and isolated from their social group, results showed that pigs, like other animals [19,20], were able to positively perceive and interact with the human after having observed a conspecific demonstrator receive long-term gentle handling. Observer pigs showed greater affiliative behaviors and lower stress levels (as indicated by the lower low/high frequency ratio) toward the stockperson compared to control pigs. However, the reaction of observer pigs toward the stockperson was not modulated by the social rank of the demonstrator conspecific. In our study, there were no differences in the social learning performance of observer pigs after being exposed to socially dominant and subordinate demonstrators. Animals from both treatment groups behaved similarly when they were individually confronted by the stockperson. Additionally, our study showed a sexual dimorphism in the physiological response to stress during the interaction with the stockperson. Specifically, the females were better adapted to confrontation with the human in an unfamiliar environment compared to male pigs.

### 4.1. Behavioral Response of Observers during the Interaction with the Stockperson in the Open-Field Arena Test

When observer pigs were evaluated in an unfamiliar environment, no differences were found between animals exposed to socially dominant and subordinate demonstrators. Unlike control animals, the observer pigs exhibited greater affiliative behaviors toward the stockperson during all phases, even in those phases where the stockperson adopted a threatening posture for pigs, such as remaining in a standing up position or approaching and trying to stroke them [8,47]. The greater affinity shown by observer pigs was characterized by lower latencies to first contact and to entry in the stockperson area and by animals staying in close proximity with the stockperson for a longer period of time compared to control animals. Similar affiliative behaviors toward humans have been reported in lambs [4,48], cows [3,49] and pigs [5,8,11,36] exposed to regular gentle contact (e.g., stroking, brushing), indicating a positive HAR [2]. According to Rault et al. [50], the acceptance of approach and subsequent stroking by a human are clear indicators of a positive HAR. In our study, observer pigs showed a reduced avoidance response toward the stockperson when she tried to stroke them (Phase 4), resulting in a higher percentage of accepted strokes compared to control pigs. Moreover, fewer attempts were required by the stockperson to complete the first stroke in comparison to controls, which means that observer pigs were able to recognize the stroking provided to their demonstrator as non-threatening stimuli. These results differ from those reported by de Oliveira et al. [36], who did not observe differences in the acceptance of physical contact (i.e., accepted strokes) between non-handled piglets (control group) and piglets (observers) that were previously exposed to daily presence of a human in the pen while performing forced tactile contacts on their littermates. These differences with our study could be explained by the imposed nature of tactile interactions used in the study of Oliveira et al. [36], who performed forced strokes to piglets, irrespective of whether the animals showed resistance or emitted vocalizations during the first minute of handling. Even if the tactile stimulation was carried out in a positive and gentle way, observer piglets might have initially detected some distress in their handled litter mates. This, consequently, might have caused a conditioned negative perception of the human handling (stroking), through emotional contagion, eliciting the observer piglets to behave similarly to controls.

When animals are confronted with novel and unexpected stimuli (social or non-social), they may respond with behaviors motivated by both curiosity and fear [51]. In the present study, most of the behaviors displayed by the control animals were likely triggered primarily by fear. Unlike the observer pigs, the control pigs were distressed, as indicated by the increase in time spent looking at the stockperson [11,52], the increased amount of low-pitched [46] and high-pitched vocalizations emitted [53,54], and the occurrence of eliminative behaviors (defecation and urination) during the interaction with the stockperson [55]. All of these behaviors have been observed in pigs exposed to stressful and negative situations [56], such as introduction to a novel environment [57], isolation from conspecifics [53,57,58] and confrontation with a human in a human-approach test [11,55,57]. In addition to the behaviors previously mentioned, the greater latencies to approach and to contact the stockperson, the longer time they spent away from the stockperson and the increased avoidance of physical contact (e.g., greater locomotor activity when the stockperson tried to stroke them and less accepted strokes) suggests that control pigs were not just fearful of a new environment but of the human as well [55]. These results are in line with previous studies that have shown that pigs receiving a minimal amount of human contact, limited to routine husbandry, are highly fearful of humans compared to pigs exposed to a regular positive contact [8,11] or to daily human presence inside the pen [8,52].

Social learning abilities have been well-established in the case of fear response, where animals can learn fearful behaviors by watching how their conspecifics respond to a stimulus. This form of social learning is often described as observational conditioning [17]. For instance, Rhesus monkeys can acquire a fear for snakes after having observed a conspecific behaving fearfully when confronted with a snake [59,60]. However, to elucidate whether farm animals have the ability to learn to positively perceive the human, through genuine observational social learning, has been the main questioning that previous studies on this topic have received [19,20,21]. A valid criticism of these studies is that it is possible that social learning had no effect at all and that animals were instead habituated to the human presence, regardless of the positive nature of handling being observed by animals. In pigs, both regular presence of a motionless human in the home pen and being regularly stroked are equally effective in increasing approach behaviors to the human, compared to a minimal human-pig contact [8]. However, Brajon et al. [8] showed that when pigs were subjected to a human-approach test (comparable to the moving human phase in the present study), pigs that were exposed to human presence without being stroked (habituation group) did not show any differences from those that received minimal contact (control group); both groups exhibited a greater avoidance response when the experimenter actively attempted to touch them. However, pigs that received strokes showed lower reactivity to the experimenter and accepted to be touched [8]. In contrast, in our study we found that observer pigs accepted more stroking in comparison to controls, and surprisingly, up to 70% of them accepted being touched and stroked at the first attempt. This could indicate that beyond a simple habituation to the stockperson presence inside the pen, pigs learned to associate the human and the handling provided to their demonstrators with positive experiences despite never receiving positive tactile contacts throughout the experimental procedure. Therefore, analogous to studies in Rhesus monkeys, pigs could have learned to not fear the human by watching gently handled demonstrators behaving calmly in response to the stockperson during the GH sessions. However, despite the fact that our results suggest effective observational learning when they are compared with those of Brajon et al. [8], the only way to determine the validity of the criticism to the studies previously reported is to conduct experiments that include a group of animals visually exposed to a human presence inside the pen without physical interaction. On the other hand, even though these results can be attributed to observational social learning, the effects of other senses (such as smell, touch or taste) during the GH procedure of the demonstrator pig cannot be discarded, and neither the influence of the interactions between penmates after the demonstrator pig has returned to its social group post GH procedure. Therefore, future studies are required to further investigate these potential effects.

### 4.2. Physiological Response to the Interaction with the Stockperson in the Open-Field Arena Test

The LH/HF ratio is one of the most commonly HRV measures used to assess the balance between the autonomic nervous system branches, where greater values are associated with a predominance of sympathetic activity and, consequently, with higher levels of physical and psychological stress [38,41]. When the physiological response was analyzed, animals from the control group exhibited an increase of relative values of LF and the LF/HF ratio, indicating higher stress levels in this group in comparison to the other treatment groups. These results are in concordance with the behavioral reactions of fear displayed by control animals during the open-field test.

In our study, no change in indicators of parasympathetic activity (i.e., HF, RMSSD) were found between treatment groups. The root mean square of successive inter-beat intervals is an indicator that reflects the vagal influence, which is known to predominate at rest and under positive affective states [38]. The lack of parasympathetic activity predominance in DDG and SDG pigs compared to control pigs may have been caused by locomotor activity displayed in the open-field environment, in which animals could explore and move freely, thus changing the autonomic balance toward sympathetic predominance. On the other hand, isolation from their groupmates might have prevented observer pigs from experiencing positive emotions with the stockperson and from showing changes in RMSSD as a consequence. Therefore, the absence of changes in RMSSD is likely caused by both factors. However, our results are in line with Imfeld-Mueller et al. [61], who did not find differences in the RMSSD when pigs were confronted with a positive situation compared to a negative one. Furthermore, similar results have been reported in other species, such as dogs [62] and goats [63], where the RMSSD did not increase while animals were experiencing a positive situation.

Sexual dimorphism in the behavioral and physiological responses to stress has been documented in several species [64], including pigs [57,58,65]. Situations that are stressful or potentially threatening for males are not necessarily stressful for females, and the reverse. In pigs, there is evidence that certain HRV measures are affected in a sex-specific manner after exposure to a stressor [51]. Our results showed that female and male pigs physiologically responded differently during the interaction with the stockperson. In the present study, males showed an increase in HR and SDNN, and a greater decrease in RMSSD/SDNN ratio, with no change in RMSSD. This indicates a shift of the autonomic balance toward a sympathetic predominance [38,66], suggesting that males were more vulnerable to social stress in the presence of a human than female pigs. In addition, the males exhibited a greater increase in the relative values of LF and LF/HF ratio than females. Taken together, these results suggest that female pigs could be better adapted to social challenges, such as the confrontation with the human in an unfamiliar environment, while the males would be more susceptible to stress under the same challenging conditions. These results are in accordance with Zupan et al. [51], who found that barrows socially isolated in a novel environment exhibited a greater increase in LF/HF ratio than gilts, indicating a higher stress susceptibility in this group. Moreover, our results are in line with Reimert et al. [57], who showed that castrated male piglets were more susceptible to stress and responded more fearful to a human-approach test than female piglets, having even higher basal cortisol concentrations than females [57]. On the other hand, the greater physiological adaptability shown by females can be emphasized by the behavioral response observed when the stockperson remained sitting and motionless (Phase 3). In this phase, females showed a greater willingness to climb on the stockperson, suggesting a greater risk-taking propensity and a clear search for more intensive contact with the human compared to male pigs [11].

### 4.3. Effect of Social Rank of Demonstrator Pig on Observer Pigs’ Response in the Open-Field Arena Test

Previous studies have reported a bias in social learning, in which the individuals preferentially attend to and copy the behavior of higher rather than lower ranked conspecifics [67,68,69]. For instance, Nicol and Pope [70] found that social learning of a keypeck response to obtain food was greater in observer laying hens when a socially dominant hen was used as demonstrator than when a subordinate hen was used. In addition, most correct keypecks were made by observer hens that had seen dominant demonstrators perform the task before [70]. However, no differences in the efficiency of information transmission between higher and lower social ranked demonstrators were found in the current study. Indeed, observer pigs exposed to socially dominant and subordinate demonstrators reacted similarly when they were confronted with the human in a non-familiar environment after watching the positive handling of their demonstrators. The finding that neither demonstrator pig was more efficient than the other suggests that a positive handling of previously selected demonstrators, regardless of their social position within the group, could be a useful strategy to reduce fear levels in animals (observers), at least when they are individually confronted with the stockperson in an unfamiliar environment (e.g., during a procedure involving temporary separation from the group). However, it has been reported that socially learned behaviors by an individually tested observer (i.e., in the absence of their groupmates) may not necessarily be expressed in a population assessed under natural conditions (i.e., animals within their social group) [71]. Therefore, considering pig husbandry practices under commercial production, further investigation is required to assess possible differences in learning performance of pigs when they are confronted by a human in a familiar environment (rearing pen with their groupmates). The assessment of animals under a familiar context would allow to determine if during the interaction between the human and the observer pigs, the presence of a socially dominant (or subordinate) demonstrator affects the observer pigs´ reaction and motivation to interact with the human.

## 5. Conclusions

Providing pigs with the opportunity to observe a conspecific demonstrator being positively handled by the stockperson leads to the acquisition of a positive perception of the human through observational social learning. This could have meaningful relevance in intensive swine production systems, where the stockpersons only have a limited amount of time to positively interact with the animals. The positive handling of previously selected demonstrators could be a useful strategy to reduce the level of fear in a larger group of animals. In addition, the study indicates that there were no differences in social learning performance after the animals were exposed to socially dominant and subordinate demonstrators. Observer pigs from both treatment groups show similar motivation to interact with the human in an unfamiliar environment, in absence of the demonstrator pig and penmates.

## Figures and Tables

**Table 1:** Schedule of the experiment.

Day of the Experiment	Pigs Age (Days)	Event/Test	Place	Measurements
1	21	Weaning	Commercial farm	-
1–7	21–27	Acclimation to nursery pens	Home pen	-
1–2	21–22	Agonistic behaviors register	Home pen	Behavioral
3–5	23–25	Agonistic behaviors analysis	-	Behavioral
6	26	Dominance Index estimation	-	Dominance index
8–40	28–60	Gentle handling sessions	Home pen	-
27–28	47–48	Habituation to band of Polar device	Home pen	-
29–31	49–51	Habituation to arena and transport cage	Open-field arena	-
34–35	54–55	Basal HRV measurement	Home pen	Baseline HRV
44–46	64–66	Open-field arena test	Open-field arena	Behavioral and HRV

HRV = heart rate variability.

**Table 2:** Behavioral observations evaluated during the interaction with the stockperson in each phase of the open-field arena test.

Behavioral Observation	Description	Phase
Latency to first physical contact	Time (s) taken for the pig to make physical contact with any part of body of the stockperson	2, 3
Latency to approach the area around the stockperson	Time (s) taken for the pig to enter an area within 0.5 m diameter around the point at which the stockperson stood	2, 3
Time of contact	Time (%) that pig stayed in physical contact with any part of body of the stockperson, either touching or sniffing it	2, 3
Time in area around the stockperson	Time (%) that pig spent within 0.5 m diameter around of the stockperson, either in contact with her (touching or sniffing) or while exploring the environment	2, 3
Looking at the stockperson	Time (%) that pig stood still looking at the stockperson, with its body and head oriented toward the stockperson in attention attitude	2, 3
Frequency of contact	Number of times the pig physically contacted the stockperson	2, 3
Climbing on the stockperson	Frequency of climbing on the stockperson, with at least the front legs on the thighs, and number of animals that were observed climbing on the stockperson	3
Accepted strokes	Strokes (%) accepted by each pig based on the total attempts made by the stockperson	4
Attempts required until accepting the first stroke	Number of attempts made by the stockperson until the pig accepted the first stroke	4
High-pitched vocalizations	Number of screams, squeals and grunt-squeals	1, 2, 3, 4
Low-pitched vocalizations	Number of short or long grunts	1, 2, 3, 4
Locomotor activity	Number of zones that the pig crossed with its front legs	1, 2, 3, 4
Defecation	Number of animals that were observed defecating in any phase where the stockperson was present	2, 3, 4
Urination	Number of animals that were observed urinating in any phase where the stockperson was present	2, 3, 4

**Table 3:** Pigs behavioral responses during the different phases of the open-field arena test according to their treatment.

Behavior		Treatments		*p*-Value
	CG	DDG	SDG	
	(*n* = 24)	(*n* = 20)	(*n* = 20)	
Phase 1				
Locomotion	35.83 ± 3.20	39.45 ± 3.39	34.40 ± 3.39	0.571 ^1^
Low-pitched vocalization	20.66 ± 3.78	29.40 ± 4.14	19.45 ± 4.14	0.183 ^1^
High-pitched vocalization	0 (0–0)	0 (0–0)	0 (0–0)	0.562 ^3^
Phase 2				
Locomotion	11.34 ± 1.80	9.00 ± 1.88	8.80 ± 1.88	0.567 ^1^
Low-pitched vocalization	16.92 ± 2.74	14.05 ± 2.94	12.60 ± 2.94	0.561 ^1^
High-pitched vocalization	0 (0–0.75)	0 (0–3.5)	0 (0–0)	0.152 ^3^
Latency to approach area (s)	42.66 ± 4.24 ^a^	19.18 ± 4.81 ^b^	18.89 ± 3.66 ^b^	0.001 ^2^
Latency to first contact (s)	48.22 ± 3.83 ^a^	31.34 ± 5.30 ^b^	22.62 ± 3.98 ^b^	0.001 ^2^
Time looking at the stockperson (%)	47.43 ± 6.47 ^a^	23.30 ± 6.80 ^b^	22.29 ± 6.80 ^b^	0.027 ^1a^
Time in area (%)	20.14 ± 6.49 ^a^	45.77 ± 7.11 ^b^	45.39 ± 7.11 ^b^	0.002 ^1a^
Time of contact (%)	8.28 ± 4.79 ^a^	27.12 ± 5.25 ^b^	25.60 ± 5.25 ^b^	0.003 ^1a^
Physical contact (frequency)	0.74 ± 0.35 ^a^	1.85 ± 0.36 ^b^	2.15 ± 0.36 ^b^	0.039 ^1^
Phase 3				
Locomotion	21.98 ± 2.33 ^p^	13.95 ± 2.40 ^q^	14.50 ± 2.40 ^q^	0.058 ^1^
Low-pitched vocalization	29.71 ± 5.19 ^p^	14 ± 5.45 ^q^	15 ± 5.45 ^q^	0.100 ^1^
High-pitched vocalization	0 (0–2.75) ^p^	0 (0–0) ^q^	0 (0–0) ^q^	0.080 ^3^
Latency to approach area (s)	42.21 ± 8.49 ^a^	4.09 ± 1.47 ^b^	9.07 ± 3.53 ^b^	<0.001 ^2^
Latency to first contact (s)	47.11 ± 8.97 ^a^	5.47 ± 1.64 ^b^	10.02 ± 3.65 ^b^	<0.001 ^2^
Time looking at the stockperson (%)	13.30 (3.18–28.05) ^a^	1.60 (0–8.35) ^b^	1.04 (0–5.87) ^b^	0.001 ^3^
Time in area (%)	45.93 ± 7.42 ^a^	84.19 ± 7.82 ^b^	77.11 ± 7.82 ^b^	0.008 ^1a^
Time of contact (%)	37.64 ± 8.35 ^a^	76.35 ± 8.68 ^b^	65.30 ± 8.68 ^b^	0.014 ^1a^
Physical contact (frequency)	3.96 ± 0.78	3.75 ± 0.80	3.99 ± 0.80	0.975 ^1^
Climb on the stockperson (frequency)	1.10 ± 0.54	1.65 ± 0.56	0.84 ± 0.56	0.592 ^1^
Phase 4				
Locomotion	37.90 ± 2.53 ^a^	28.62 ± 2.69 ^b^	23.22 ± 2.69 ^b^	0.005 ^1^
Low-pitched vocalization	33.50 ± 3.49 ^a^	17.10 ± 3.77 ^b^	12.70 ± 3.77 ^b^	0.003 ^1^
High-pitched vocalization	2 (0–12.50) ^a^	0 (0–0) ^b^	0 (0–0) ^b^	0.007 ^3^
Accepted strokes (%)	25.36 ± 5.92 ^a^	80.51 ± 6.24 ^b^	84.77 ± 6.35 ^b^	<0.001 ^1a^
Attempts until first accepted stroke	5.50 (4–8.50) ^a^	1 (1–2) ^b^	1 (1–2) ^b^	<0.001 ^3^

Values within a row with different letters significantly differ (^a^,^b^: *p* < 0.05) or tend to differ (^p^,^q^: 0.05 < *p* < 0.1). CG = control group; DDG = demonstrator dominant group; SDG = subordinate demonstrator group. Phase 1 = Habituation to arena; Phase 2 = Standing stationary person; Phase 3 = Sitting stationary person; Phase 4 = Moving person. ^1^ mixed ANOVA model; ^1a^ mixed ANOVA based on angular transformation (df = 2); ^2^ Kaplan–Meier analysis (df = 2); ^3^ Kruskal–Wallis test (df = 2). Data analyzed with ANOVA mixed models are expressed as estimated marginal means and standard error means (E.M.M. ± S.E.M.) of non-transformed data. Data analyzed with the Kaplan–Meier method are expressed as means and standard error (m ± S.E.) and data analyzed with Kruskal–Wallis test are expressed with medians and interquartile ranges (md (Q25–Q75)).

**Table 4:** Estimated marginal means ± S.E.M. of the relative values (open field test—baseline = Δ) of heart rate variability parameters of pigs from different treatments and sexes during the interaction with the stockperson in the open-field test.

Parameter (Unit)		Treatments			Sex		
	CG	DDG	SDG	*p*-Value	Female	Male	*p*-Value
	(*n* = 21)	(*n* = 20)	(*n* = 19)		(*n* = 30)	(*n* = 30)	
RR Interval (ms)	−30.02 ± 8.58	−13.13 ± 8.17	−24.24 ± 8.32	0.358 ^1^	−13.44 ± 6.43 ^p^	−31.49 ± 6.42 ^q^	0.057 ^1^
HR (bpm)	14.05 ± 4.28	6.57 ± 4.08	11.65 ± 4.15	0.465 ^1^	7.04 ± 3.21 ^p^	14.47 ± 3.20 ^q^	0.100 ^1^
SDNN (ms)	10.49 ± 1.81	5.72 ± 1.87	6.46 ± 1.84	0.190 ^1^	5.31 ± 1.41 ^a^	9.81 ± 1.42 ^b^	0.020 ^1^
RMSSD (ms)	2.71 ± 0.83	1.33 ± 0.87	1.68 ± 0.85	0.506 ^1^	1.51 ± 0.66	2.31 ± 0.66	0.379 ^1^
RMSSD/SDNN (ms)	−0.11 ± 0.04	−0.08 ± 0.04	−0.10 ± 0.04	0.874 ^1^	−0.05 ± 0.03 ^a^	−0.14 ± 0.03 ^b^	0.034 ^1^
LF (ms)	505.27 ± 67.49 ^a^	118.76 ± 69.57 ^b^	109.48 ± 68.30 ^b^	0.003 ^1^	127.20 ± 52.09 ^a^	361.80 ± 52.28 ^b^	0.001 ^1^
HF (ms)	96.76 ± 25.36	89.79 ± 26.66	89.53 ± 26.02	0.975 ^1^	72.14 ± 21.24	111.92 ± 21.24	0.191 ^1^
LF/HF (ms)	2.023 ± 0.83 ^a^	−2.14 ± 0.85 ^b^	−0.91 ± 0.84 ^b^	0.015 ^1^	−1.56 ± 0.63 ^a^	0.87 ± 0.63 ^b^	0.004 ^1^

Values within a row with different letters significantly differ (^a^,^b^: *p* < 0.05) or tend to differ (^p^,^q^: 0.05 < *p* < 0.1). CG = control group; DDG = dominant demonstrator group; SDG = subordinate demonstrator group. RR Interval = intervals between adjacent heart beats; HR = heart rate; SDNN = standard deviation of the inter-beat intervals; RMSSD = the root mean square of subsequent inter-beat intervals; LF = low frequency band; HF = high frequency band. ms = millisecond; bpm = beats per minute. ^1^ mixed ANOVA model. Data are expressed as estimated marginal means and standard error means (E.M.M. ± S.E.M.).

## Data Availability

The data presented in this study are available from the corresponding author on reasonable request.
